# Extreme Preterm Delivery Between 24^+0^ and 27^+6^ Weeks: Factors Affecting Perinatal Outcome

**DOI:** 10.3390/jcm14041064

**Published:** 2025-02-07

**Authors:** Joanna Kowalczyk-Buss, Eleftheria Demertzidou, Sara El-Toukhy, Ghada Ramadan, Ranjit Akolekar

**Affiliations:** 1 Medway Fetal and Maternal Medicine Centre, Medway NHS Foundation Trust, Kent ME7 5NY, UK; 2 Department of Obstetrics and Gynaecology, Institute of Mother and Child, 01-211 Warszawa, Poland; 3 Nottingham Medical School, University of Nottingham, Nottingham NG7 2RD, UK; 4 Oliver Fisher Neonatal Unit, Medway NHS Foundation Trust, Kent ME7 5NY, UK; 5 Institute of Medical Sciences, Canterbury Christ Church University, Canterbury CT1 1QU, UK

**Keywords:** preterm delivery, extreme prematurity, neonatal death, perinatal survival

## Abstract

**Objectives:** The aim of this study was to investigate the factors associated with the prediction of perinatal survival in pregnancies with extreme preterm delivery between 24^+0^ and 27^+6^ weeks’ gestation. **Methods:** This screening cohort study was undertaken at a large tertiary obstetric and neonatal unit in the United Kingdom. We included singleton pregnancies that booked and delivered at our hospital. Logistic regression analysis was carried out to determine risks of complications in pregnancies delivering preterm after adjusting for maternal and pregnancy characteristics. Effect sizes were expressed as absolute risks (ARs) and odds ratios (ORs) (95% confidence intervals [CI]). **Results:** The study population included 53,649 singleton pregnancies, including 139 (0.3%) with preterm delivery between 24^+0^ and 27^+6^ weeks and 47,006 (99.7%) with term delivery ≥37 weeks. Multivariate regression analysis demonstrated that there was a significant contribution of uterine artery pulsatility index (UtA-PI) and cervical length, but not of maternal factors, in the prediction of preterm delivery <28 weeks. The risk of neonatal death and intact neurological survival in pregnancies delivering <28 weeks was 11.5% and 79.1%, respectively. Caesarean compared to vaginal delivery and female compared to male neonates were associated with a lower incidence of neurological morbidity (6.1% vs. 19.3%; *p* = 0.016 and 13.1% vs. 26.9%; *p* = 0.036, respectively). In the prediction of intact perinatal survival, the only significant variable was gestational age at delivery, with survival rates of about 50%, 65%, 80% and 90% at 24, 25, 26 and 27 weeks, respectively. **Conclusions:** In pregnancies with extreme preterm delivery between 24^+0^ and 27^+6^ weeks, caesarean compared to vaginal delivery and female compared to male neonates are associated with a lower incidence of neurological morbidity. The only significant factor in the prediction of intact perinatal survival is gestational age at delivery.

## 1. Introduction

Extreme preterm delivery is defined as delivery prior to 28 completed weeks of gestation [1]. There is evidence from studies demonstrating that extreme prematurity is associated with significantly increased risk of perinatal complications including intraventricular haemorrhage (IVH), hypoxic ischaemic injury (HII), respiratory distress syndrome (RDS), jaundice, hypoglycaemia, and neonatal death (NND) [2,3,4,5]. However, there is considerable variation in the literature with regard to total and intact neurological survival in pregnancies that deliver extremely preterm; for instance, survival at 25 weeks’ gestation ranged from 59% in the EPIPAGE 2 study [6], 67% in the EPICURE study [7,8] and 76% in the NICHD study [9] to 82% in the EXPRESS study [10]. In addition, these studies reported results based on data from more than 10–15 years ago and therefore do not necessarily reflect the standards of current neonatal care. Recent data from the United Kingdom published on behalf of the MBRRACE-UK collaboration on the survival of babies born before 27 weeks’ gestation states that total neonatal survival is 5% at 22 weeks’, 71% at 25 weeks’ and 80% at 26 weeks’ gestation, which is significantly improved compared to data from the EPICURE study [11].

The objectives of our study were, first, to examine the maternal and pregnancy characteristics associated with extreme preterm delivery between 24^+0^ and 27^+6^ weeks’ gestation and to investigate the independent contribution of maternal factors, biomarker values such as pregnancy-associated plasma protein-A (PAPP-A), uterine artery pulsatility index (UtA-PI) and cervical length in the prediction of preterm delivery; second, to compare the incidence of neonatal complications in those delivering preterm compared to those that deliver at term and derive estimates of total and intact perinatal survival for each gestational week; and third, to undertake a stratified analysis of complications based on the mode of delivery, indications for preterm delivery and foetal gender to examine which factors affect neurological morbidity and intact survival.

## 2. Materials and Methods

### 2.1. Study Population

This was a retrospective cohort study undertaken at Medway Fetal and Maternal Medicine Centre, United Kingdom, between January 2010 and December 2022 in a large, unselected population that booked their pregnancy care at our hospital prior to 14 weeks’ gestation during the study period. At our hospital, all women are offered a scan attended at 11–13 weeks’ gestation for the dating of the pregnancy, combined screening for foetal aneuploidies and systematic examination of the foetal anatomy [12,13,14]. The next assessment is at 20–22 weeks’ gestation for the examination of foetal growth and anatomy, placental localisation, uterine artery Doppler to assess impedance to blood flow across the placenta and cervical length measurement [15,16]. All pregnancies are offered a risk assessment for iatrogenic and spontaneous preterm delivery based on an assessment of maternal factors, obstetric and gynaecology history, cervical length assessment and uterine artery Doppler [15,17,18]. Those that are deemed to be at high chance for spontaneous prematurity are referred to the preterm birth clinic at 14–15 weeks’ gestation and those at risk of iatrogenic preterm delivery due to placental insufficiency are referred to the placental disorder clinic.

Data regarding maternal demographic characteristics, obstetric and medical history, ultrasound findings and pregnancy outcomes were recorded on an electronic database (Viewpoint version 5.6; GE Healthcare, Buckinghamshire, UK). The protocol for this study was reviewed by the National Research Ethics Committee and approved by the Health Research Authority in the United Kingdom (REC reference number 24/HRA/0660). The inclusion criteria for this study were singleton pregnancies that delivered between 24^+0^ and < 28 weeks’ gestation and those that delivered at term (≥37 weeks’ gestation). We excluded pregnancies that had a miscarriage or stillbirth, those with multiple pregnancies, foetuses with major structural or genetic defects and those that were lost to follow-up.

### 2.2. Outcome Measures

The outcome measures that we examined were admission to the Neonatal Care Unit (NCU) and total stay in intensive care or high-dependency unit (ITU and HDU, respectively), NND, IVH, HII, hypoglycaemia, RDS, jaundice and intact neonatal survival. IVH was defined according to the Papile criteria [19]. HII was defined as evidence of hypoxic injury on brain imaging including non-cystic white matter injury and periventricular leukomalacia [20]. Hypoglycaemia was defined by neonatal serum glucose level <2.6 mmol/L [21]. RDS was defined as inability to maintain adequate oxygen saturations with spontaneous respirations, demonstrations of clinical signs such as grunting, flaring, tachypnoea or intercoastal retractions requiring additional respiratory support [22]. Neonatal jaundice was based on visual inspection and the observation of a yellow discoloration of the skin or sclera coupled with increased blood serum total bilirubin measurement [23]. Intact neurological survival was defined as survival at discharge in the absence of any abnormal neurological findings such as IVH or HII.

### 2.3. Statistical Analysis

Data were expressed as median (interquartile range) for continuous variables and as *n* (%) for categorical variables. Comparison between groups for continuous variables was by Mann–Whitney U-test for continuous variables and by the χ^2^-square test or Fisher’s exact test for categorical variables. A significant difference between groups was assumed at 5% and post hoc Bonferroni correction was used for any multiple comparisons.

Prior to regression analysis, continuous variables such as maternal age, weight, height and BMI were centred around the mean to avoid effects of multicollinearity. Univariate logistic regression analysis was carried out for each adverse outcome to derive odds ratios (ORs) with 95% confidence intervals (CIs). Multivariate logistic regression analysis with backwards stepwise elimination was then carried out to determine which of the maternal and pregnancy factors provided a significant contribution in the prediction of iatrogenic and spontaneous preterm delivery < 28 weeks. Pregnancies with extreme prematurity were stratified according to mode of delivery, indication for delivery and neonatal gender, and the risk of neonatal complications within each stratified group was compared with term deliveries (≥37 weeks’ gestation), and a comparison between groups within the preterm cohort was also performed. The predicted probabilities derived from regression analysis were used to derive estimates of risk of HII, neonatal death and intact neonatal survival for each day between 24^+0^ and < 28 weeks’ gestation. The statistical package SPSS 29.0 (IBM SPSS Statistics for Windows, Version 24.0, Armonk, NY, USA, IBM Corp; 2022) was used for data analyses.

## 3. Results

### 3.1. Study Population

During the study period, 53,649 women with singleton pregnancies were booked for delivery at our hospital. We excluded 1929 pregnancies (3.6%) that were lost to follow-up, 581 (1.1%) that had a miscarriage at <24 weeks, 168 (0.3%) that had stillbirths, 669 (1.3%) with major foetal defects and 3157 (5.9%) that delivered between 28.0 and 36.6 weeks’ gestation; the study population was therefore formed of 47,145 singleton pregnancies, including 139 (0.3%) with preterm delivery < 28 weeks and 47,006 (99.7%) controls with term delivery ≥37 weeks. There were 23 (16.5%) pregnancies delivered for iatrogenic indications and 116 (83.5%) that delivered spontaneously.

### 3.2. Maternal and Pregnancy Characteristics

The maternal and pregnancy characteristics in the study population are shown in Table 1. The incidence of preterm delivery < 28 weeks’ gestation was significantly higher in those with a BMI > 35, a history of cigarette smoking in pregnancy and South Asian racial origin. The incidence of chronic hypertension, pre-existing diabetes mellitus and pre-eclampsia was higher in those with a preterm delivery compared to those that delivered at term. In pregnancies with preterm delivery, compared to those delivered at term, the median PAPP-A MoM and cervical length measurement were lower, whereas the uterine artery PI MoM was higher. The rate of those with a low-PAPP-A MoM <0.3 (5.0% vs. 1.6%; *p* = 0.001), UtA-PI ≥ 95th percentile (26.0% vs. 6.4%; *p* < 0.001) and cervical length <25 mm (20.5% vs. 2.7%; *p* < 0.001) was significantly higher in the preterm delivery group compared to those that delivered at term. There were no significant differences with regard to maternal age, height or method of conception, nor in foetal CRL, NT, free β-hCG MoM at 11–13 weeks or EFW percentile at 20–22 weeks’ assessment (Table 1).

Multivariate regression analysis demonstrated that, in the prediction of iatrogenic preterm delivery < 28 weeks, there was a significant independent prediction from chronic hypertension (OR: 6.98; 95% CI: 2.06–23.64; *p* = 0.002), parity with previous history of delivery of SGA neonate (OR: 6.26; 95% CI: 1.99–19.69; *p* = 0.002) and UtA-PI ≥ 95th percentile (OR: 12.21; 95% CI: 5.15–28.96; *p* < 0.001). Similarly, in the prediction of spontaneous preterm delivery, there was a significant contribution of maternal age (OR: 0.95; 95% CI: 0.92–0.98; *p* = 0.005), BMI (OR: 1.05; 95% CI: 1.02–1.08; *p* = 0.001), South Asian racial origin (OR: 3.06; 95% CI: 1.54–6.05; *p* = 0.001), cigarette smoking (OR: 1.81; 95% CI: 1.15–2.86; *p* = 0.011), UtA-PI ≥ 95th percentile (OR: 2.79; 95% CI: 1.72–4.54; *p* < 0.001), SUA (OR: 3.37; 95% CI: 1.22–9.34; *p* = 0.019), previous history of miscarriage 16–23 weeks (OR: 3.67; 95% CI: 1.19–11.30; *p* = 0.024) and cervical length < 25 mm (OR: 9.29; 95% CI: 5.46–15.79; *p* < 0.001).

### 3.3. Perinatal Adverse Outcomes

In pregnancies with preterm delivery < 28 weeks compared to term deliveries, as expected, there was a significant increase in neonatal adverse outcomes (Table 2). The median total stay in NCU was 68 days for preterm deliveries, compared to 3 days for those delivered at term. Similarly, admission to HDU/ITU was associated with a median stay of 38 days for preterm neonates as opposed to 1 day for term neonates. There was a significantly increased risk of IVH, HII, hypoglycaemia, RDS, jaundice and neonatal death in preterm compared to term neonates. The rate of NND in term neonates was 0.01% compared to 11.5% in preterm neonates (*p* < 0.001). The rate of intact neurological survival was 79.1% in the preterm group compared to 99.8% in the term group (OR 0.008; 95% CI: 0.005–0.013) (*p* < 0.001) (Table 2).

### 3.4. Factors Affecting Perinatal Outcomes

**Figure 1:**
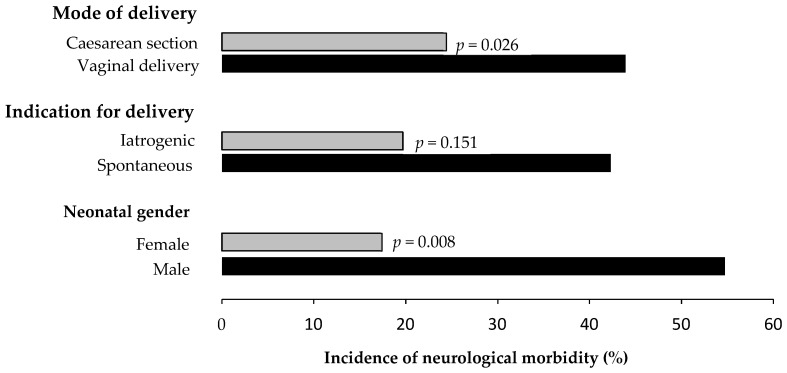
Incidence of neurological morbidity stratified by mode of delivery, indication for delivery and neonatal gender.

In the analysis of the preterm delivery group stratified by mode of delivery, the rate of major neurological morbidity was significantly lower in the caesarean section (CS) group compared to the vaginal delivery (VD) group [24.4% (20/82) vs. 43.9% (25/57); *p* = 0.026), with a higher rate of intact perinatal survival (86.6 vs. 70.2%; *p* = 0.030). There were no significant differences in the rates of other adverse outcome in the CS and VD groups (Table 3). The rates of major neurological morbidity in preterm deliveries stratified by indication for delivery demonstrated that the rate in those delivered for iatrogenic indications was 17.4% (4/23) compared to 54.7% (41/75) if the delivery was spontaneous, but this difference did not reach statistical significance (*p* = 0.151). All 23 (100%) neonates in the iatrogenic group were delivered by CS, at a median gestation of 27.0 (IQR: 26.1–27.3) weeks, whereas 59 (50.9%) in the spontaneous group were delivered by CS, at a median gestation of 26.1 (IQR: 25.2–27.2) weeks. There were no significant differences in other outcomes measures based on indication for delivery (Table 4). The rate of major neurological morbidity was significantly lower in female neonates compared to male neonates [19.7% (12/61) vs. 42.3% (33/78); *p* = 0.008). There were no significant differences in other neonatal adverse outcomes between male and female neonates (Table 5). The rates of major neurological morbidity in pregnancies delivering < 28 weeks stratified by mode of delivery, indication for delivery and neonatal gender are shown in Figure 1.

### 3.5. Prediction of Adverse Perinatal Outcome

**Figure 2:**
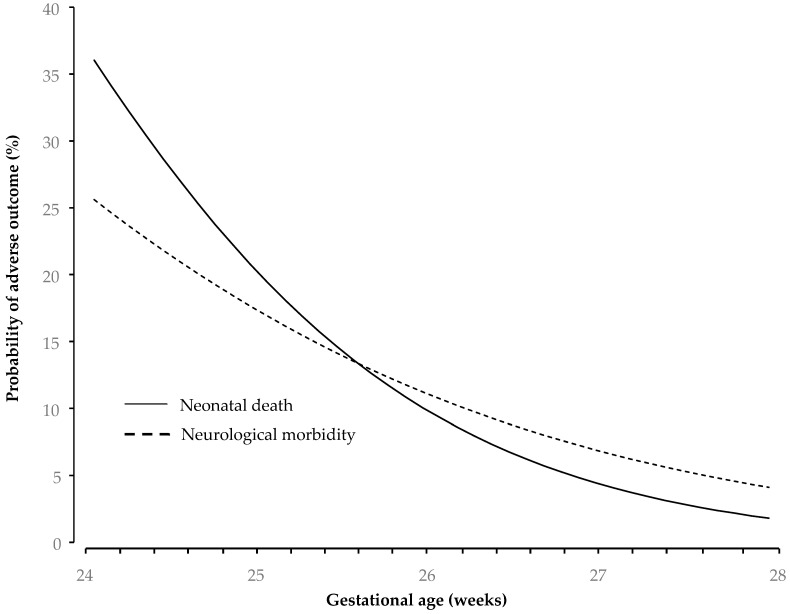
Association between risk of NND and major neurological morbidity with gestational age at delivery.

Logistic regression analysis demonstrated that there was a significant linear association between risk of NND and major neurological morbidity with gestational age at delivery (Figure 2), which was expressed as follows:

Y = 19.547 + (−0.838 × gestational age at delivery); −2 Log likelihood = 88.24, Nagelkerke R2 = 0.149; model *p* value < 0.001; where Y is the probability of NND.

Z = 11.378 + (−0.518 × gestational age at delivery); −2 Log likelihood = 94.71, Nagelkerke R2 = 0.063; model *p* value < 0.001; where Z is the probability of neurological morbidity.

**Figure 3:**
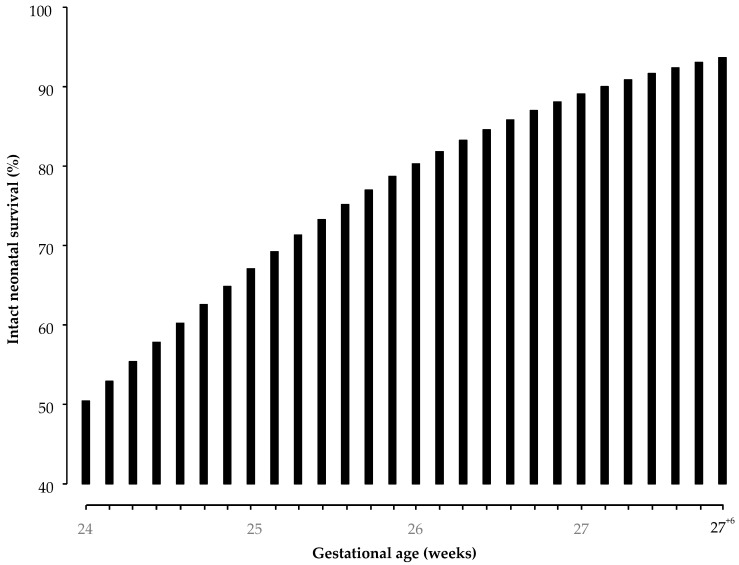
The probability of intact perinatal survival for each gestational day from 24 weeks to 27^+6^ weeks.

Similarly, multivariate regression analysis demonstrated that in the prediction of neurologically intact perinatal survival in pregnancies delivering < 28 weeks’ gestation, there was a significant contribution of gestational age at delivery (*p* < 0.001) but not of maternal factors such as maternal age (*p* = 0.444), BMI (*p* = 0.171), racial origin (*p* = 0.874), cigarette smoking (*p* = 0.611), IVF conception (*p* = 0.397), parity (*p* = 0.382), PAPP-A MoM (*p* = 0.538), uterine artery PI (*p* = 0.155), EFW (*p* = 0.943) and cervical length (*p* = 0.939), nor from mode of delivery (*p* = 0.849), indication for delivery (*p* = 0.548) or foetal gender (*p* = 0.711). The predominant factor predicting intact survival was gestational age at delivery and the prediction was defined by the equation:

Y= −16.634 + 0.694 (95% CI:0.291–1.097) × gestational age at delivery; −2 Log likelihood = 129.85, Nagelkerke R2 = 0.134; model *p* value <0.001, where Y is the probability of intact neurological survival. The intact neurological survival rates at 24, 25, 26, 27 and 28 weeks were 50.5%, 67.1%, 80.3%, 89.1% and 94.2%, respectively. The probability of intact perinatal survival for each gestational day from 24 weeks to 27^+6^ weeks is outlined in Table 6 and Figure 3.

## 4. Discussion

### 4.1. Principal Findings of This Study

The main findings of our study demonstrate the following: first, the incidence of preterm delivery < 28 weeks is 0.3%; second, multivariate regression demonstrates that the factors which predict an iatrogenic preterm delivery include chronic hypertension, history of delivery of SGA neonate and uterine artery PI > 95th percentile, whereas factors providing a significant prediction of spontaneous preterm delivery include maternal age, BMI, south Asian racial origin, cigarette smoking, uterine artery PI > 95th percentile, history of miscarriage at 16–23 weeks and cervical length < 25 mm; third, the rate of major neurological morbidity is significantly lower in pregnancies delivering by CS compared to those that deliver vaginally, with a correspondingly higher rate of intact neurological survival in the CS group; fourth, the risk of major neurological morbidity in female neonates is lower compared to male neonates; and lastly, the only factor providing a significant prediction of intact neurological survival in multivariate regression based on maternal and pregnancy factors is gestational age at delivery, with a rate of 50% at 24^+0^ weeks to 94% at 27^+6^ weeks.

### 4.2. Comparison with Other Studies

This incidence of extreme preterm delivery between 24^+0^ and 27^+6^ weeks in our study was 0.3%. This is similar to other studies, including a large population-based study based in Sweden which reported a similar incidence in their cohort (0.3%; 1011/305,318) [10,24]. The results of our study demonstrate that the rate of intact neurological survival in pregnancies with preterm delivery between 24^+0^ and 27^+6^ weeks is 79.1% (95% CI: 71.6–85.1). There is a linear relationship between intact survival and gestational age, with rates of 50.4%, 67.1%, 80.3%, 89.1% and 93.7% at 24, 25, 26, 27 and 27^+6^ weeks’ gestation, respectively. This is consistent with recent data reported in the British Association for Paediatric Medicine (BAPM) framework for practice, which states that the total perinatal survival in babies admitted to the NCU is about 45%, 63%, 77% and 84% at 23, 24, 25 and 26 weeks’ gestation, respectively [25]. Our results are also consistent with a meta-analysis of 27 studies reporting survival in extreme prematurity stated that perinatal survival was 53.9% for deliveries at 24 weeks, increasing to 90.1% at 27 weeks’ gestation [26]. Similarly, in a Swedish population study of 1011 extremely preterm neonates delivered before 27 weeks in 305,318 pregnancies, the authors reported that the rate of survival was 71% at 24 weeks and 87% at 27 weeks’ gestation. The rate of caesarean deliveries in this Swedish cohort of deliveries between 24 and 27 weeks was 60%, which is similar to that noted in our series (59%), implying that there may be a potential impact of the mode of delivery on perinatal survival [10]. The rate of major neurological morbidity noted in our cohort stratified by mode of delivery demonstrated a significantly lower rate in pregnancies delivered by CS (24.4%) compared to VD (43.9%), with a corresponding higher rate of intact neurological survival in pregnancies delivered by CS. There are several studies which report that the rate of perinatal mortality in pregnancies delivering vaginally is higher compared to those delivering by CS [27,28,29]. In a study by Hogberg et al., the authors reported that in pregnancies delivering between 23^+0^ and 27^+6^ weeks’ gestation, the rate of perinatal mortality was significantly lower in the CS group (25.8%) compared to those delivering vaginally (31.6%) [28]. There is evidence in the literature that the rate of preterm delivery and its associated complications is higher in male neonates compared to female neonates [25,30,31]. In a large population-based study from Japan based on 1,098,268 singleton pregnancies, the authors reported that male foetal gender compared to female gender was associated with a significantly increased risk of all types of preterm delivery, including extreme prematurity [31]. In our study, there was a trend towards a higher prevalence of male compared to female gender in preterm deliveries (56.1% vs. 43.9%). We also noted that the risk of major neurological adverse outcomes associated with extreme preterm delivery is lower in female neonates compared to male neonates (19.7% vs. 42.3%).

The results of our study provide estimates of total and intact perinatal survival at various gestational ages in extreme preterm deliveries between 24^+0^ to 27^+6^ weeks’ gestation which are consistent with those reported in other larger population-based studies. In addition, we investigate the association of intact perinatal survival with maternal demographic characteristics, pregnancy and ultrasound findings, as well as factors such as mode of delivery, indication for preterm delivery, foetal gender and gestational age, which demonstrates that the single most important factor that impacts perinatal outcomes is gestational age at delivery. There is a linear relationship between intact perinatal survival and gestational age at delivery which provides estimates of survival at each day and week and which would be useful for healthcare professionals and families.

### 4.3. Strengths and Weaknesses of the Study

The strengths of our study are, first, the examination of a large consecutive cohort of more than 50,000 consecutively screened pregnancies who booked at 11–13 weeks’ gestation with available data for maternal and neonatal outcomes; second, pregnancies managed in a tertiary unit for foetal medicine and obstetrics with available data on maternal demographic characteristics, biomarkers such as PAPP-A, uterine artery PI and cervical length; third, management of pregnancies and neonates in a tertiary Level 3 neonatal intensive care unit that routinely manages neonates delivering at extreme preterm gestations; and fourth, the use of multivariable regression analysis to investigate the independent contribution of maternal factors, pregnancy findings and biomarker values within the preterm cohort but also to undertake a stratified analysis to examine whether the mode of delivery, indication for delivery and foetal gender affect the prediction of adverse perinatal outcomes in extreme prematurity. The limitation our study is that this is a study on singleton pregnancies and the estimates for risks in multiple pregnancies may be different. This is a single-centre study from a tertiary foetal and neonatal unit and estimates of risks in other settings may be different. A limitation of this study is that we have reported short-term neonatal outcomes, as long-term data and outcomes were unavailable to the study team. Despite this study being based on a single centre, our results should be applicable to any tertiary-level foetal and neonatal unit which provides management to pregnancies at risk of iatrogenic and spontaneous preterm deliveries. Despite our data being based on tertiary level management, they will provide useful information to those providing care in settings with limited resources to allow for sharing these risks with patients and families and arrange for timely in utero transfer of women to centres that can provide specialist care. Our results are focused on immediate perinatal mortality and morbidity and should provide clinicians and patients with latest and contemporary evidence-based estimates of risks of complications in extreme prematurity to make decisions regarding the mode and timing of delivery. Further studies are required to investigate longer-term risks of morbidity.

## 5. Conclusions

The incidence of preterm delivery < 28 weeks is 0.3%. In current clinical practice for pregnancies managed in a tertiary obstetric and neonatal unit, the chance of total and intact perinatal survival is about 90% and 80%, respectively. The main factor which dictates the outcome in extreme prematurity is gestational age at delivery, which has an inverse linear association with the risk of neonatal death and a linear association with the chance of intact perinatal survival. Our study provides estimates of risk of neonatal death, neurological morbidity and intact perinatal survival for each day between 24^+0^–27^+6^ weeks’ gestation, which may assist healthcare professionals and families in decision making.

## Figures and Tables

**Table 1:** Maternal demographic and pregnancy characteristics in pregnancies with preterm delivery compared to term delivery stratified by gestational age at delivery.

Maternal Demographics	Delivery ≥37 Weeks (*n* = 47,006)	Preterm Delivery <28 Weeks (*n* = 139)	*p* Value
Maternal age in years, median (IQR)	29.1 (25.1–32.9)	29.0 (23.5–32.2)	0.136
Maternal BMI in kg/m2, median (IQR)	25.4 (22.3–29.9)	27.1 (23.0–32.4)	0.007
BMI < 18, *n* (%)	1318 (2.8)	3 (2.2)	0.645
BMI > 35, *n* (%)	4694 (10.0)	24 (17.3)	0.004
Racial origin			
Caucasian, *n* (%)	42,713 (90.9)	118 (84.9)	0.022
Afro-Caribbean, *n* (%)	1491 (3.2)	8 (5.8)	0.083
South Asian, *n* (%)	2046 (4.4)	11 (7.9)	0.040
Other, *n* (%)	756 (1.6)	2 (1.4)	0.613
In vitro fertilisation, *n* (%)	717 (1.5)	3 (2.2)	0.543
Cigarette smoking, *n* (%)	7004 (14.9)	35 (25.2)	<0.001
Medical disorders			
Chronic hypertension, *n* (%)	476 (1.0)	5 (3.6)	0.002
Diabetes mellitus, type 1 or 2, *n* (%)	308 (0.7)	4 (2.9)	0.014
Obstetric complications			
Gestational hypertension, *n* (%)	768 (1.6)	3 (2.2)	0.626
Preeclampsia, *n* (%)	948 (2.0)	9 (6.5)	<0.001
11–13 weeks’ scan			
Foetal CRL (mm), median (IQR)	65.3 (59.7–71.5)	64.8 (60.3–70.1)	0.511
Foetal NT (mm), median (IQR)	1.8 (1.5–2.1)	1.9 (1.6–2.1)	0.131
Free β-hCG MoM, median (IQR)	1.01 (0.69–1.52)	0.92 (0.67–1.32)	0.083
PAPP-A MoM, median (IQR)	1.00 (0.70–1.43)	0.85 (0.56–1.40)	0.016
PAPP-A < 0.3 MoM, *n* (%)	737 (1.6)	7 (5.0)	0.001
20–22 weeks’ scan			
EFW percentile, median (IQR)	56.3 (31.6–78.9)	56.2 (20.5–80.6)	0.484
EFW < 10th percentile, *n* (%)	2824 (6.1)	16 (11.5)	0.007
Uterine artery PI > 95th centile, *n* (%)	2883 (6.4)	34 (26.0)	<0.001
Cervical length (mm), median (IQR)	33 (30–37)	31 (26–35)	<0.001
Cervical length < 25 mm, *n* (%)	759 (2.7)	18 (20.5)	<0.001
Birth weight in grams, median (IQR)	3454 (3140–3772)	875 (750–1030)	<0.001
Birth weight percentile, median (IQR)	54.1 (27.4–78.2)	56.4 (16.8–84.9)	0.932
Gestation at delivery, median (IQR)	39.6 (39.0–40.6)	26.2 (25.3–27.2)	<0.001

IQR: interquartile range; CRL: crown–rump length; NT: nuchal translucency; hCG: human chorionic gonadotropin; PAPP-A: pregnancy-associated plasma protein-A; EFW: estimated foetal weight.

**Table 2:** Neonatal adverse outcomes in pregnancies with preterm delivery <28 weeks compared to those that delivered at term expressed as absolute risks and odds ratios (ORs) with 95% confidence intervals (CIs).

Neonatal Adverse Outcomes	Delivery ≥37 Weeks (*n* = 47,006)	Preterm Delivery < 28 Weeks (*n* = 139)	
		Median (IQR) or *n* (%)	OR (95%CI)
Total NCU stay ^a^	3 (2–5)	68 (40–80) *	1.19 (1.17–1.22)
ITU/HDU stay ^a^	1 (0–2)	38 (21–57) *	1.48 (1.42–1.55)
IVH	5 (0.01)	29 (20.9; 15.0–28.4) *	2478.24 (941.95–6520.12)
HII	95 (0.2)	16 (11.5; 7.1–18.0) *	64.23 (36.74–112.28)
Hypoglycaemia	325 (0.7)	26 (18.7; 13.0–26.1) *	33.05 (21.28–51.32)
RDS	829 (1.8)	105 (75.5; 67.7–82.0) *	172.02 (116.14–254.78)
Jaundice	1503 (3.2)	97 (69.8; 61.7–76.8) *	69.92 (48.51–100.79)
Neonatal death	8 (0.01)	16 (11.5; 7.1–18.0) *	764.20 (321.14–1818.49)
Intact survival	46,905 (99.8)	110 (79.1; 71.6–85.1) *	0.008 (0.005–0.013)

^a^ Data presented as median (interquartile range). Mann–Whitney U test for continuous variables and χ^2^ test or Fisher’s exact test for categorical variables. Significance level * *p* < 0.01. NCU = neonatal intensive care unit; ITU = intensive care unit; HDU = high-dependency unit; IVH = intraventricular haemorrhage; HII = hypoxic ischaemic injury; RDS = respiratory distress syndrome.

**Table 3:** Neonatal adverse outcomes in pregnancies with preterm delivery <28 weeks compared to those that delivered at term expressed as absolute risks and odds ratio (ORs) with 95% confidence intervals (CIs), stratified by mode of delivery.

Neonatal Adverse Outcomes	Delivery ≥37 Weeks (*n* = 47,006)	Preterm Delivery < 28 Weeks			
		Caesarean Delivery (*n* = 82)		Vaginal Delivery (*n* = 57)	
		Median (IQR) or *n* (%)	OR (95%CI)	Median (IQR) or *n* (%)	OR (95%CI)
Total NCU stay ^a^	3 (2–5)	64 (23–75) **	1.18 (1.15–1.21)	74 (64–92) *	1.19 (1.16–1.23)
ITU/HDU stay ^a^	1 (0–2)	32 (16–49) **	1.44 (1.35–1.53)	45 (26–64) *	1.48 (1.40–1.58)
IVH	5 (0.01)	15 (18.3; 11.3–28.1) **	2104.5 (743.8–5954.7)	14 (24.6; 15.1–37.2) *	3060.5 (1056.1–8869.2)
HII	95 (0.2)	5 (6.1; 2.3–13.8) **	32.1 (12.7–81.0)	11 (19.3; 11.0–31.5) *^,†^	118.1 (59.4–235.0)
Hypoglycaemia	325 (0.7)	11 (13.4; 7.5–22.6) **	22.3 (11.7–42.4)	15 (26.3; 16.6–39.1) *	51.3 (28.2–93.4)
RDS	829 (1.8)	63 (76.8; 66.6–84.7) **	184.7 (110.1–310.0)	42 (73.7; 60.9–83.4) *	156.0 (86.2–282.4)
Jaundice	1503 (3.2)	59 (72.0; 61.4–80.6) **	77.7 (47.8–126.1)	38 (66.7; 53.7–77.6) *	60.6 (34.8–105.3)
Neonatal death	8 (0.01)	8 (9.8; 4.8–18.3) **	635.1 (232.2–1737.1)	8 (14.0; 7.0–25.6) *	959.1 (346.1–2657.9)
Intact survival	46,905 (99.8)	71 (86.6; 77.4–92.5) **	0.011 (0.006–0.021)	40 (70.2; 57.3–80.5) *^,†^	0.006 (0.003–0.010)

^a^ Data presented as median (interquartile range) in days; * = comparison of preterm delivery with term delivery; ^†^ = comparison within preterm delivery groups between caesarean and vaginal delivery. Mann–Whitney U test for continuous variables and χ^2^ test or Fisher’s exact test for categorical variables. Adjusted significance level after post hoc Bonferroni correction for multiple comparisons, * *p* < 0.0167, ** *p* < 0.001. NCU = neonatal care unit; ITU = intensive care unit; HDU = high-dependency unit; IVH = intraventricular haemorrhage; HII = hypoxic ischaemic injury; RDS = respiratory distress syndrome.

**Table 4:** Neonatal adverse outcomes in pregnancies with preterm delivery < 28 weeks compared to those that delivered at term ≥37 weeks, stratified by iatrogenic and spontaneous delivery.

Neonatal Adverse Outcomes	Delivery ≥37 Weeks (*n* = 47,006)	Preterm Delivery <28 Weeks			
		Iatrogenic Delivery (*n* = 23)		Spontaneous Delivery (*n* = 116)	
		Median (IQR) or *n* (%)	OR (95%CI)	Median (IQR) or *n* (%)	OR (95%CI)
Total NCU stay ^a^	3 (2–5)	55 (12–69) **	1.19 (1.16–1.22)	71 (41–82) *	1.21 (1.16–1.26)
ITU/HDU stay ^a^	1 (0–2)	33 (12–46) **	1.49 (1.41–1.57)	40 (22–59) *	1.48 (1.37–1.61)
IVH	5 (0.01)	3 (13.0; 3.7–33.0) **	1410.0 (315.8–6300.2)	26 (22.4; 15.7–30.9) *	2715.6 (1020.0–7229.7)
HII	95 (0.2)	1 (4.3; 0.10–22.7) **	22.5 (3.0–168.2)	15 (12.9; 7.9–20.4) *	73.3 (41.1–130.8)
Hypoglycaemia	325 (0.7)	3 (13.0; 3.7–33.0) **	21.6 (6.4–72.9)	23 (19.8; 13.5–28.1) *	35.5 (22.2–56.8)
RDS	829 (1.8)	20 (87.0; 67.0–96.3) **	371.5 (110.1–1252.1)	85 (73.3; 64.5–80.5) *	152.7 (100.7–231.7)
Jaundice	1503 (3.2)	19 (82.6; 62.3–93.6) **	148.8 (48.9–423.2)	78 (67.2; 58.3–75.1) *	62.1 (42.0–91.9)
Neonatal death	8 (0.01)	3 (13.0; 3.7–33.0) **	881.2 (217.9–3564.3)	13 (11.2; 6.5–18.4) *	741.5 (301.0–1826.8)
Intact survival	46,905 (99.8)	19 (82.6; 62.3–93.6) **	0.01 (0.003–0.031)	91 (78.4; 70.1–85.0) *	0.008 (0.005–0.011)

^a^ Data presented as median (interquartile range) in days; * = comparison of preterm delivery with term delivery. Mann–Whitney U test for continuous variables and χ^2^ test or Fisher’s exact test for categorical variables. Adjusted significance level after post hoc Bonferroni correction for multiple comparisons, * *p* < 0.0167, ** *p* < 0.001. NCU = neonatal care unit; ITU = intensive care unit; HDU = high-dependency unit; IVH = intraventricular haemorrhage; HII = hypoxic ischaemic injury; RDS = respiratory distress syndrome.

**Table 5:** Neonatal adverse outcomes in pregnancies with preterm delivery < 28 weeks compared to those that delivered at term ≥37 weeks, stratified by neonatal gender.

Neonatal Adverse Outcomes	Delivery ≥37 Weeks (*n* = 47,006)	Preterm Delivery <28 Weeks			
		Male Neonates (*n* = 78)		Female Neonates (*n* = 61)	
		Median (IQR) or *n* (%)	OR (95%CI)	Median (IQR) or *n* (%)	OR (95%CI)
Total NCU stay ^a^	3 (2–5)	68 (33–82) **	1.19 (1.16–1.22)	70 (40–80) *	1.21 (1.16–1.26)
ITU/HDU stay ^a^	1 (0–2)	43 (21–60) **	1.49 (1.41–1.57)	35 (22–57) *	1.48 (1.37–1.61)
IVH	5 (0.01)	21 (26.9; 18.3–37.7) **	3463.2 (1262.3–9502.0)	8 (13.1; 6.5–24.1) *	1418.9 (449.6–4478.3)
HII	95 (0.2)	12 (15.4; 8.9–25.2) **	89.8 (47.0–171.5)	4 (6.6; 2.1–16.1) *	34.7 (12.3–97.4)
Hypoglycaemia	325 (0.7)	12 (15.4; 8.9–25.2) **	26.1 (14.0–48.8)	14 (23.0; 14.1–35.0) *	42.8 (23.3–78.5)
RDS	829 (1.8)	60 (76.9; 66.4–85.0) **	185.7 (109.2–315.8)	45 (73.8; 61.5–83.3) *	156.7 (88.2–278.3)
Jaundice	1503 (3.2)	55 (70.5; 59.6–79.5) **	72.4 (44.4–118.1)	42 (68.9; 56.4–79.1) *	66.9 (38.8–115.3)
Neonatal death	8 (0.01)	8 (10.3; 5.1–19.2) **	671.4 (245.1–1839.0)	8 (13.1; 6.5–24.1) *	886.8 (320.9–2450.1)
Intact survival	46,905 (99.8)	61 (78.2; 67.8–86.0) **	0.008 (0.004–0.014)	49 (80.3; 68.5–88.5) *	0.009 (0.005–0.017)

^a^ Data presented as median (interquartile range) in days; * = comparison of preterm delivery with term delivery. Mann–Whitney U test for continuous variables and χ^2^ test or Fisher’s exact test for categorical variables. Adjusted significance level after post hoc Bonferroni correction for multiple comparisons, * *p* < 0.0167, ** *p* < 0.001. NCU = neonatal care unit; ITU = intensive care unit; HDU = high-dependency unit; IVH = intraventricular haemorrhage; HII = hypoxic ischaemic injury; RDS = respiratory distress syndrome.

**Table 6:** Probability of neurological morbidity, neonatal death and intact neurological survival according to gestational age at delivery.

Gestational Age (Weeks and Days)	Neurological Morbidity (%)	Neonatal Death (%)	Intact Survival (%)
24^+0^	25.7	36.1	50.4
24^+1^	24.4	33.4	52.9
24^+2^	23.0	30.7	55.5
24^+3^	21.7	28.2	57.8
24^+4^	20.5	25.9	60.2
24^+5^	19.3	23.7	62.5
24^+6^	18.1	21.5	64.9
25^+0^	17.1	19.6	67.1
25^+1^	16.1	17.8	69.2
25^+2^	15.1	16.1	71.4
25^+3^	14.2	14.5	73.3
25^+4^	13.3	13.1	75.2
25^+5^	12.5	11.9	76.9
25^+6^	11.7	10.6	78.7
26^+0^	10.9	9.5	80.3
26^+1^	10.3	8.6	81.8
26^+2^	9.6	7.6	83.3
26^+3^	8.9	6.8	84.6
26^+4^	8.4	6.1	85.8
26^+5^	7.8	5.5	87.0
26^+6^	7.3	4.9	88.1
27^+0^	6.8	4.4	89.1
27^+1^	6.4	3.9	90.0
27^+2^	5.9	3.5	90.9
27^+3^	5.5	3.1	91.7
27^+4^	5.2	2.7	92.4
27^+5^	4.8	2.5	93.0
27^+6^	4.5	2.2	93.7

## Data Availability

The original contributions presented in the study are included in the article, further inquiries can be directed to the corresponding authors.
